# The Role of Inflammasomes in Heart Failure

**DOI:** 10.3390/ijms25105372

**Published:** 2024-05-14

**Authors:** Panayotis K. Vlachakis, Panagiotis Theofilis, Ioannis Kachrimanidis, Konstantinos Giannakopoulos, Maria Drakopoulou, Anastasios Apostolos, Athanasios Kordalis, Ioannis Leontsinis, Konstantinos Tsioufis, Dimitris Tousoulis

**Affiliations:** 1st Department of Cardiology, “Hippokration” General Hospital, National and Kapodistrian University of Athens, 11527 Athens, Greece; vlachakispanag@gmail.com (P.K.V.); panos.theofilis@hotmail.com (P.T.); iskachrimanidis@gmail.com (I.K.); giannakw79@gmail.com (K.G.); mdrakopoulou@hotmail.com (M.D.); anastasisapostolos@gmail.com (A.A.); akordalis@gmail.com (A.K.); giannisleontsinis@gmail.com (I.L.); kptsioufis@gmail.com (K.T.)

**Keywords:** inflammasomes, heart failure, inflammation, biomarkers, therapeutic modulation

## Abstract

Heart failure (HF) poses a significant world health challenge due to the increase in the aging population and advancements in cardiac care. In the pathophysiology of HF, the inflammasome has been correlated with the development, progression, and complications of HF disease. Discovering biomarkers linked to inflammasomes enhances understanding of HF diagnosis and prognosis. Directing inflammasome signaling emerges as an innovative therapeutic strategy for managing HF. The present review aims to delve into this inflammatory cascade, understanding its role in the development of HF, its potential role as biomarker, as well as the prospects of modulating inflammasomes as a therapeutic approach for HF.

## 1. Introduction

Heart failure (HF) constitutes a significant global health challenge due to the rise in the aging population and advancements in cardiac care, which extend the lives of heart disease patients. The extent of the HF “problem” remains challenging to ascertain accurately due to the absence of reliable, population-based data on its prevalence, incidence, and prognosis [1]. Variations in diagnostic criteria and the growing use of pre-symptomatic left ventricle (LV) dysfunction as an indicator further complicate assessment [2]. Globally, an estimated 56 million individuals worldwide have HF [3]. 

Despite therapeutic advancements, mortality rates among HF patients have remained notably elevated [4]. In recent years, there has been significant advancement in the understanding of HF pathophysiology, transitioning from viewing it solely as a hemodynamic disorder to recognizing it as a complex systemic disease with multiple factors [5,6]. Alongside the acknowledged involvement of the sympathetic nervous system (SNS) and the renin–angiotensin–aldosterone system (RAAS) as a fundamental aspect in both the pathophysiology and management of the syndrome, the role of inflammation has been extensively investigated for many years [7]. In HF patients, inflammation has been associated with the development, progression, and complications of the disease [8]. Additionally, it serves as a predictive factor for unfavorable outcomes, irrespective of conventional metrics such as LV ejection fraction or New York Heart Association (NYHA) class [9]. Advancements in basic research offer the potential to discover new targets for the treatment and prevention of HF. One such target on the horizon is inflammatory signaling, which is partly mediated by inflammasomes, multiprotein complexes primarily expressed by immune cells [10].

Given the potential role of inflammasomes in HF pathophysiology and their potential as therapeutic targets and biomarkers, the aim of this review paper is to delve into this inflammatory cascade, with particular emphasis on exploring the physiological roles of inflammasomes, their involvement in the pathophysiology of HF, their potential as biomarkers, and the prospects of modulating inflammasomes as a therapeutic approach for HF. 

## 2. Physiology of Inflammasomes

Inflammasomes, critical components of the innate immune system, are complex molecular structures primarily found in immune cells such as macrophages and dendritic cells. They function as internal sensors and receptors, recognizing various danger signals including pathogen-associated molecular patterns (PAMPs) and damage-associated molecular patterns (DAMPs) [11]. These signals are detected by pattern recognition receptors (PRRs), which categorize microbial structures as PAMPs and endogenous danger molecules as DAMPs [12]. These patterns, including peptidoglycan, lipoteichoic acid, and lipopolysaccharides, can initiate and sustain inflammatory reactions. Numerous cell types, both immune and non-immune, utilize inflammasomes to defend against injury or infection by triggering the production of anti-inflammatory or pro-inflammatory cytokines [13].

PRRs are categorized into five main families based on their homologous protein domains: Toll-like receptors (TLRs), absent in melanoma 2 (AIM2)-like receptors, NOD-like receptors (NLRs), retinoic acid inducible gene-I (RIG-1)-like receptors, and C-type lectin receptors. While TLRs and C-type lectin receptors function as transmembrane PRRs, monitoring the extracellular space, AIM2-like receptors, RIG-I-like receptors, and NLRs serve as cytoplasmic PRRs, surveilling the intracellular space. Upon activation by PAMPs or DAMPs, some of these intracellular PRRs oligomerize and form inflammasomes [14]. Inflammasome activation involves a two-step process. The initial signal prompts the transcription and synthesis of individual components, while the subsequent signal facilities their polymerization and assembly into an active inflammasome [15]. The first signal occurs following the recognition and binding of PAMPs and DAMPs to innate immune cell receptors. This binding triggers intracellular signal transduction pathways, culminating in the activation of the cytoplasmic transcription nuclear factor kappa-light-chain-enhancer of activated B cells (NF-κB) [16]. NF-kB then promotes the transcription of genes encoding pro-interleukin (IL)-18 and pro-IL-1β cytokines, which subsequently polymerize and activate the NOD-like receptor P3 (NLRP3) [16,17]. Secondary signals, such as potassium efflux and the generation of oxygen free radicals, contribute to the polymerization and connection of the remaining components (apoptosis-associated speck-like protein containing a CARD (ASC) and procaspase-1), leading to inflammasome formation and activation. Active caspase-1, produced as a result, cleaves pro-IL-18 and pro-IL-1β into IL-18 and IL-1β, respectively, which are then secreted into the surrounding environment [18].

IL-18 and IL-1β further stimulate both non-specific and specific immune responses. The production of IL-18 and IL-1β is associated with the induction of insulin resistance tissues, which is crucial for combating infections [16]. This local insulin resistance conserves glucose and free fatty acids, essential energy substrates for immune cells fighting pathogen invasion [19]. Caspase-1, activated by inflammasome activation, along with caspase-11, cleaves gasdermin-D (GSDMD), leading to pore formation and cell death, a process known as *pyroptosis*. Consequently, the release of DAMPs occurs, potentially enhancing immunity by activating neighboring cell inflammasomes [20]. Overall, the inflammasome serves as a critical regulator of non-specific immunity, aiming to eliminate infectious invaders while limiting tissue damage through interleukin production and the autophagy pathway.

## 3. Inflammasomes in the Pathophysiology of Heart Failure

Extensive research has depicted the correlation of HF progress and the mechanisms that lead to the overexpression of biologically active molecules. These include primarily the activation of SNS, RAAS, and the natriuretic peptide system, which are involved with cardiac repair and remodeling [21]. Concerning the inflammatory mediators, there is increasing evidence about their role in the development of HF [22]. The current knowledge suggests that a myocardial injury activates the innate and adaptive immune system in the myocardial cells. The chain of events, as described in Section 2, which inflammasomes signalize, is therefore activated. This leads to a short-term adaptation of the heart cells to stress. 

Various PRRs are implicated in inflammasome formation, including NLRP3, NLRC4, and AIM2. The activation of these receptors triggers caspace-1 and IL-1/IL-18 activation, playing a crucial role in immune defense against infections [23]. In a murine model of type 2 diabetes mellitus (T2DM), myocardial infarction led to a reduction in LV ejection fraction. Mitophagy impairment in these mice resulted in the release of mitochondrial DNA, activating the AIM2 inflammasome and NLRC4 inflammasome in cardiomyocytes and macrophages within the peri-infarct region of the LV. Consequently, the activated inflammasomes and caspase-1 led to increased cell death, elevated IL-18 expression, impaired neovascularization, and enhanced fibrosis [24,25]. AIM2 expression was upregulated in the hearts of streptozotocin-induced diabetic rats, and silencing AIM2 mitigated pyroptosis, cardiac remodeling, and heart dysfunction [24,26]. Moreover, AIM2 and NLRC4 expression was elevated in the heart tissue of HF patients and animal models during the late phase of chronic HF induced by pressure or volume overload, as well as following infarction. Activation of the AIM2 inflammasome led to the activation of both IL-1 and IL-18, and its inhibition with probenecid ameliorated chronic HF [27]. These findings suggest the involvement of AIM2 and NLRC4 in diabetes-related or late-phase HF. 

Activation of the NLRP3 inflammasome has been identified as key driver of cardiac hypertrophy in response to pressure overload [28]. Under such conditions, S-nitrosylation of muscle LIM protein (MLP) facilitates the formation of a complex involving TLR-3 and receptor-interacting protein kinase 3 (RIP3). This complex activation subsequently triggers NLRP3 inflammasome activation and IL-1β production, fostering myocardial hypertrophy [29]. Studies employing pharmacologic blockade or ribonucleic acid (RNA) interference targeting NLRP3, as well as the inhibition of IL-1β with neutralizing antibodies, have demonstrated mitigated pressure overload-induced myocardial hypertrophy [30]. 

When this inflammatory response is perpetuated, the inflammation becomes chronic, inducing changes not only in cardiac myocytes but also in nonmyocytes, mainly expressed as fibrosis [31]. The NLRP3 inflammasome, in particular, has been implicated in promoting fibrosis progression primarily through the stimulation of IL-1β and IL-18 production [32]. Inhibiting NLRP3 with MCC950 has been shown to suppress myocardial-infarction-induced inflammasome activation, consequently ameliorating cardiac inflammation and fibrosis, and enhancing cardiac function [33]. Additionally, chronic activation of β-adrenergic receptors (ARs) in a pressure overload model, along with direct acute β-AR activation, induces cardiac fibrosis in an NLRP3 inflammasome-dependent manner [34,35]. Activation of the NLRP3 inflammasome via calcium/calmodulin-dependent protein kinase IId in response to pressure overload leads to heart fibrosis and dysfunction [28]. 

**Figure 1:**
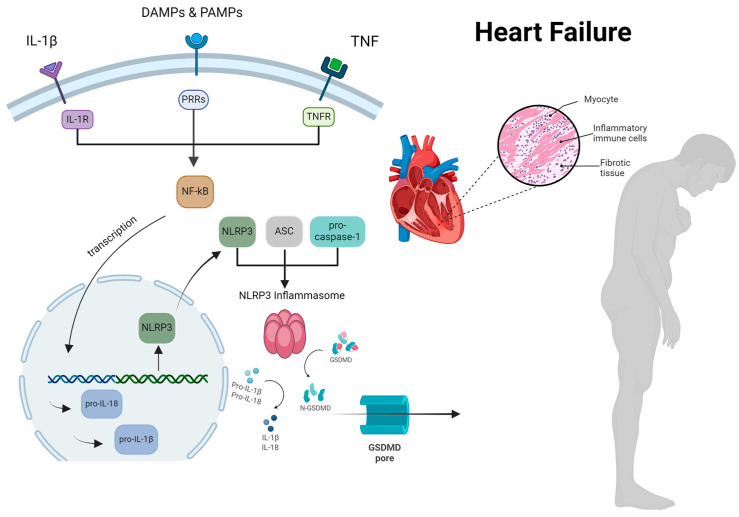
The comprehensive process by which NLRP3 inflammasome activation occurs, resulting in myocardial cell hypertrophy and inflammation in patients with heart failure. During the priming step, various stimuli such as PAMPs, DAMPs, IL-1, and TNF trigger the phosphorylation and degradation of inhibitor of NF-kB, initiating the activation of the NF-kB pathway. This pathway subsequently promotes the transcription of NLRP3, pro-IL-1, and pro-IL-18. In the activation phase, NLRP3 recruits ASC, which then binds to pro-caspase-1, facilitating the assembly of the inflammasome. The oligomeric NLRP3 inflammasome enzymatically cleaves pro-IL-1 and pro-IL-18 into their active forms, IL-1 and IL-18, respectively. Additionally, caspase-1 cleaves GSDMD, forming pores that induce pyroptosis and the release of IL-1. Created with BioRender.com. ASC, apotosis-associated speck-like protein containing a CARD; DAMP, damage-associated molecular patterns; GSDMD, gasdermin D; IL, interleukin; NF-kB, nuclear factor k-light-chain enhancer of activated B cells; NLRP3, nucleotideoligomerization domain (NOD)- like receptor P3; PRRs, pattern recognition receptors; PAMP, pathogen-associated molecular patterns; TNF, tumor necrosis factor.

In addition to fostering the activation of pro-inflammatory cytokines IL-1β and IL-18, caspase-1, activated by the NLRP3 inflammasome, instigates pyroptosis, a form of cell death characterized by the cleavage of GSDMD, resulting in the formation of pores in the cell membrane. This process exacerbates myocardial dysfunction and dilated cardiomyopathy induced by doxorubicin through NLRP3 inflammasome activation and subsequent cardiomyocyte pyroptosis [36]. Furthermore, pyroptosis amplifies inflammation by triggering the massive release of pro-inflammatory mediators upon cell death [37] (Figure 1). Notably, acute β-AR activation in cardiomyocytes has been shown to induce NLRP3 inflammasome activation and pyroptosis, with activated inflammasomes transferred to neighboring cardiac fibroblasts via membrane nanotubes in response to sympathetic overactivation, thereby exacerbating pyroptosis and inflammatory injury [38].

Additionally, there is evidence regarding the correlation between increased levels of NLRP3 inflammasome and chronic and postoperative atrial fibrillation (Afib). Studies have shown that patients with HF and Afib exhibit higher levels of IL 6, tumor necrosis factor-a (TNF-a), and C-reactive protein (CRP) compared to patients with HF without Afib [39,40]. Recent studies have also demonstrated that, among other effects, sodium glucose transport protein-2 (SGLT-2) inhibitors possess anti-inflammatory properties by reducing inflammasome activation, highlighting the significant role of inflammation in the development and progression of HF [41]. Beyond the “local“ effects of inflammasomes on the heart, their impact on the cardiovascular system as a whole in noteworthy. The association between the progression of atherosclerosis and the expression of inflammasomes is already established, yielding obvious implications for HF [42].

## 4. Inflammasomes and Their Components as Biomarkers in Heart Failure

Considering the fact that inflammasomes and their components are involved in the pathophysiology of HF, they could be potentially used as biomarkers. Previous studies have assessed the importance of NLRP3 inflammasome downstream cytokines in the incidence and severity of HF.

### 4.1. Inflammasome Components IL-1b and IL-18

Despite the potentially critical role of the NLRP3 inflammasome and its components in the pathophysiology of HF, their importance as biomarkers has not been investigated apart from in very few studies. In patients with idiopathic dilated cardiomyopathy, levels of circulating NLRP3, ASC, caspase-1, and IL-1β were significantly elevated compared to those in healthy controls, with higher NLRP3 mRNA levels correlating with reduced LV ejection fraction, and elevated natriuretic peptides and monocyte count [43]. Notably, NLRP3 and IL-1β mRNA levels at discharge emerged as independent risk factors for 6-month rehospitalization among patients and elevated NLRP3 mRNA levels were associated with an increased cumulative rehospitalization rate [43]. In the same patient population, IL-1β emerged as a strong and independent predictor of all-cause mortality, alongside male gender, atrial fibrillation, and sodium concentration [44]. IL-1β, the main downstream pro-inflammatory cytokine, has been found to be elevated in subjects with chronic HF, as shown in a previous meta-analysis [45]. Moreover, in the setting of advanced HF with the use of cardiac resynchronization therapy, IL-1β levels were predictive of the primary outcome (HF hospitalization-free survival with a decrease in end-systolic volume of at least 15%) at 12 months on univariate analysis, with the significance fading upon adjustments [46]. IL-1β may also reflect the exercise capacity of HF patients, as shown by Butts et al. in a cohort of 54 stable outpatient HF subjects [47]. Concerning acute HF, increased IL-1β levels ≥ 49.1 pg/mL were associated with incident mortality [48].

Little is known about the biomarker profile of IL-18 in HF. The study of Naito et al. has shown elevated levels in patients with HF due to previous myocardial infarction or dilated cardiomyopathy compared to non-HF groups [49]. Yamaoka–Tojo et al. showed that IL-18 concentration is higher in ischemic compared to dilated cardiomyopathy, while higher concentrations are noted in patients with a more impaired functional class [50]. IL-18 emerged to be predictive of HF hospitalization in the Atherosclerosis Risk in Communities study after multivariate adjustments [51].

### 4.2. IL-6

Inflammasome activation can lead to the release of pro-inflammatory cytokines, including IL-1β and IL-18. These cytokines can subsequently stimulate the production of IL-6 by various cell types, including immune cells and fibroblasts, as part of a broader inflammatory response. As such, studying IL-6 as an inflammasome-related biomarker in HF could be of importance, as shown in recent studies. Remmelzwaal et al. presented the findings from a case–control study investigating the association between serum IL-6 levels and new-onset HF in individuals with T2DM [52]. Results revealed that higher IL-6 concentrations are significantly correlated with increased risk of HF development in T2DM patients [52]. This association persisted across various IL-6 concentration categories and was independent of sex [52]. In another paper utilizing data from the Atherosclerosis Risk in Communities study (*n* = 5672), the association of IL-6 with HF was evaluated over a median follow-up of 7.2 years [51]. IL-6 was independently related to a 1.35-fold, 1.23-fold, and 1.37-fold higher risk of HF with preserved ejection fraction (HFpEF), HF with reduced ejection fraction, and HF hospitalization, respectively, even after adjustment for numerous confounders [51]. IL-6 was also an independent predictor of incident HF in the recently reported study of Bertero et al. after an analysis of the HUNT3 and Health ABC population cohorts [53]. Moreover, IL-6 levels above the median (≥1.2 pg/mL) in the Multi-Ethnic Study of Atherosclerosis were linked to an increased incidence of the composite of HF/cardiovascular death by 1.74-fold [54]. As far as HFpEF is concerned in particular, an analysis of 374 patients has recently highlighted its relation to a higher symptom burden, impaired exercise capacity, and excess body fat [55].

Important evidence on the role of IL-6 has also been acquired in the setting of acute HF. In one study of 2042 patients presenting to the emergency department with acute dyspnea, the vast majority of those with diagnosed HF had elevated IL-6 levels, which were highest in the setting of cardiogenic shock and lowest in hypertensive HF [56]. Critically, IL-6 levels could predict one-year mortality in this cohort, significantly improving the discriminative capability of the BIOSTAT-CHF score [56]. According to the analysis of the EDIFICA cohort, comprising 164 patients with acute HF, increased IL-6 concentration on admission was related to lower blood pressure and a greater degree of congestion [57]. Being associated with a longer in-hospital stay, higher IL-6 was predictive of HF rehospitalization and cardiovascular death, even after adjustment for renal function markers and natriuretic peptides [57]. It is important to note that such associations were not detected with CRP. Finally, Mooney et al. analyzed a cohort of 286 patients with a recent HF hospitalization and found that for each log unit increase in IL-6, the risk of all-cause death, cardiovascular death, and subsequent HF hospitalization was augmented, even after multivariable adjustment including natriuretic peptides [58].

## 5. Inflammasome Modulation in Heart Failure

### 5.1. Colchicine

Colchicine is an agent with NLRP3 inflammasome-inhibiting effects, as shown preclinically. Despite being an anti-inflammatory agent, its use in cardiovascular diseases has been proven, especially in conditions such as pericarditis and atherosclerosis [59,60]. On the other hand, evidence concerning the role of colchicine in HF is limited (Table 1). The first attempt to showcase the impact of colchicine in the setting of HF was made by Cicogna et al. in rats, by examination of the active tension and passive stiffness of LV papillary muscles before and after treatment with ascending doses of colchicine [61]. In this study, colchicine did not affect the active tension or passive stiffness of the LV papillary muscles. In another experiment, treatment with colchicine ameliorated angiotensin-II-induced apoptosis in cultured cardiomyocytes and rats, as indicated by a reduction in the caspace-3 and TUNNEL assays in vitro and in vivo [62]. In another study investigating the role of colchicine in acute myocardial infarction and ischemia–reperfusion injury, which may be responsible for HF development, there was a significant reduction in myocardial infarct size, myocardial fibrosis, and inflammatory biomarkers, which were accompanied by long-term increases in indices of cardiac output [63]. However, there was no effect on LV ejection fraction or diastolic function [63]. Contrasting results were reported in the study of Fusijue et al. in mice subjected to acute myocardial ischemia by ligation of the left anterior descending artery [64]. Treatment with colchicine for one week led to improved LV end-diastolic diameter and ejection fraction compared to the vehicle, paired with lower levels of B-type natriuretic peptide expression in the heart [64]. Moreover, the development of pulmonary edema and mortality rates were lower in the colchicine arm [64]. These findings were corroborated by reduced neutrophil and macrophage infiltration, along with downregulated mRNA expression of NLRP3 inflammasome components upon histological analysis 24 h after myocardial infarction [64]. Moving to nonischemic cardiomyopathies, colchicine was efficacious in improving indices of cardiac function (LV ejection fraction, diastolic and systolic diameter, natriuretic peptides), histological fibrosis area, circulating inflammatory biomarkers, and expression of NLRP3 inflammasome components (NLRP3, AIM2, ASC) in a murine model of doxorubicin-induced dilated cardiomyopathy [65]. The upregulation of sirtuin-2 by colchicine was postulated as the underlying mechanism in this study [65]. Finally, in a hypertension-induced HFpEF rat model, there were significant improvements in myocardial inflammation and fibrosis, which translated into ameliorated diastolic function, LV end-diastolic pressure, LV mass, and brain natriuretic peptides, among others [66]. Ultimately, an improved functional capacity and survival was noted [66]. These findings were not driven by reductions in blood pressure but rather by suppressed oxidative stress and NLRP3 inflammasome activation [66].

Clinical evidence concerning the use of colchicine in HF is scarce. The only randomized study was conducted by Deftereos et al., who enrolled patients with stable congestive HF and an LV ejection fraction ≤ 40% and administered colchicine (0.5 mg twice daily) or a matching placebo for 6 months [74]. The primary outcome of interest was an improvement in functional capacity (at least one-grade improvement in NYHA functional status) [74]. The study failed to detect a benefit in this endpoint with colchicine treatment, as well as in mortality and hospitalization rates [74]. Colchicine, however, improved LV dimensions and produced a significant decrease in inflammatory biomarkers [74]. 

The interest in the use of colchicine has resurged recently and a few studies are currently underway in HFpEF patients. The COLchicine HEART Failure PRESERVED Trial (COLHEART-PRESERVED) is designed to explore the impact of colchicine on the health status, quality of life, and vascular and cardiac function in patients diagnosed with HFpEF (NCT06081049). The trial will enroll approximately 152 patients aged 40 years and older who meet HFpEF criteria. Participants will be randomly assigned to receive either a low-dose colchicine treatment (0.5 mg once daily) or a placebo, with treatment duration lasting 6 months. Another study also aims to explore the role of low-dose colchicine in improving functional capacity, imaging indices of cardiac function, and inflammation in 60 patients with HFpEF (NCT06130059). Finally, COL-Micro-HF is an ongoing mechanistic study in 60 patients with HF and LV ejection fraction > 40%, through which the researchers aim to showcase a potential role of 6-month colchicine treatment in improving coronary microvascular dysfunction (assessed with coronary flow reserve) in this patient population (NCT06217120). 

Colchicine’s use in acute HF decompensation is also under investigation, with one study aiming to enroll such patients with increased markers of inflammation and randomize them to colchicine or placebo (0.6 mg twice daily for 14 days followed by 0.6 mg once daily for 76 days) (NCT06286423). The designated outcomes concern the changes in high-sensitivity CRP and IL-6, while the incidence of all-cause mortality and HF hospitalizations at 90 days will also be assessed. In the COLICA study of acute decompensated HF patients, changes in N-terminal pro-B-type natriuretic peptide (NTproBNP) will be examined according to treatment with colchicine and placebo after a 2-month follow-up (NCT04705987). Moreover, the investigators will test whether colchicine can lead to clinical stability, assessed with NYHA class change, diuretic dose requirements, and HF decompensation leading to hospitalization, among others.

### 5.2. MCC950

MCC950 selectively inhibits the NLRP3 inflammasome through the prevention of the oligomerization process of NLRP3, a crucial step required for the assembly of the inflammasome complex [76]. Although still being exclusively studied in the preclinical stage, several interesting observations have been made concerning its possible efficacy in HF. Initially, MCC950 was administered in mice lacking G-protein-coupled estrogen receptor on their cardiomyocytes, a model mimicking postmenopausal heart disease development. In this model, there was impaired myocardial relaxation, reduced fractional shortening, and abnormal concentrations of atrial natriuretic factor and brain natriuretic peptide [67]. Following 8 weeks of treatment with MCC950, the researchers noted attenuated hypertrophy and improvements in the aforementioned indices [67]. Next, MCC950 was administered in a mouse model of transient aortic constriction-induced pressure overload [68]. In the group of mice receiving the treatment, there was a reversal in myocardial function evidenced by improved LV ejection fraction and cardiac dimensions [68]. Notably, those changes were accompanied by attenuated cardiac hypertrophy, oxidative stress, inflammation, and fibrosis [68]. Similar findings to the previous experiment were reported in obese mice on a high-fat diet that underwent transient aortic constriction and were subsequently treated with MCC950 [77]. However, the investigators of this study additionally observed a restoration of fatty acid uptake and utilization, together with reduced glucose uptake and oxidation, signifying improved cardiac metabolism [77]. Shi et al. tried to document the impact of MCC950 in isoproterenol-induced cardiomyopathy, replicating comparable improvements in cardiac function, inflammation, oxidative stress, and fibrosis [69]. Moreover, the investigators found that treatment of H9C2 cardiomyocytes with MCC950 also led to diminished cell death and senescence as a result of oxidative stress attenuation [69]. Furthermore, MCC950 may improve pulmonary artery pressure and remodeling through inhibition of the NLRP3 inflammasome, as shown in an experimental HFpEF model by Cheng et al. [70]. Finally, MCC950 administration exhibited a reduction in susceptibility to ventricular arrhythmias in the setting of preclinical HF, as evidenced by the abbreviated QTc duration and action potential duration 90 (APD_90_), the lowered threshold for APD alternans, and the decreased rate of ventricular arrhythmia induction [71]. Moreover, the introduction of MCC950 resulted in elevated protein levels of ion channels such as Kv4.2, KChIP2, and Cav1.2 [71]. Despite the promising results of preclinical studies concerning MCC950, there are no completed or ongoing human studies to date.

### 5.3. Other NLRP3 Inflammasome Inhibitors

**Figure 2:**
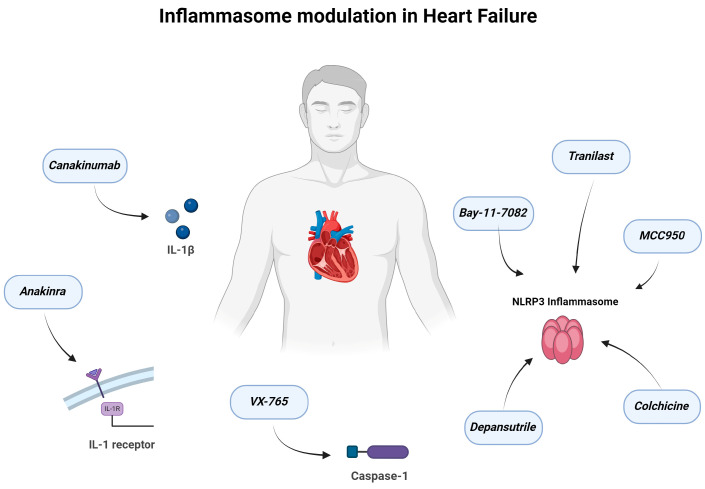
Therapeutic targets to modulate inflammasome in heart failure. Created with BioRender.com.

Several NLRP3 inflammasome inhibitors (VX765, INF39, JC124) are at various stages of preclinical development, but the evidence regarding their role in HF is currently lacking. On the contrary, the specific NLRP3 inflammasome inhibitor CY-09 was able to reverse ponatinib-induced cardiotoxicity by suppressing the inflammatory response in the study of Tousif et al. [72]. OLT1177, also known as dapansutrile, has shown promise according to a study in mice with non-reperfused ischemic cardiomyopathy [73]. Its oral administration for 9 weeks led to an improved myocardial contractile reserve at 10 weeks post myocardial infarction, together with ameliorated diastolic function [73]. Notably, a phase 1b, dose-escalation, randomized trial in patients with HFrEF and impaired functional capacity has also been performed [75]. According to its findings, patients exhibited improvements in LV ejection fraction and exercise capacity after treatment with the maximal dapansutrile dose (2000 mg) for 14 days, without any safety concerns [75]. Given those early encouraging findings, subsequent phase trials are eagerly awaited. Figure 2 provides a summary of therapeutic targets aimed at modulating inflammasomes in patients with HF. 

## 6. Conclusions

The crucial role of inflammasomes in the pathophysiology of HF cannot be understated, as they significantly contribute to myocardial inflammation, adverse remodeling, and disease progression. The identification of inflammasome-related biomarkers has emerged as a crucial aspect in HF diagnosis and prognosis, offering valuable insights into disease severity and progression. Targeting inflammasome signaling holds promise as a novel therapeutic strategy for HF management, offering the potential to mitigate myocardial inflammation and improve cardiac function. 

In moving forward, it is imperative to expand research efforts in several critical directions. Future investigations should prioritize the conduct of clinical trials aimed at evaluating the efficacy and safety of interventions targeting inflammasome pathways in HF patients. Additionally, there is a pressing need for the development of compound derivates with enhanced pharmacokinetic profiles, thereby improving the specificity and efficacy of NLRP3 inhibitors. Both preclinical and clinical research endeavors should endeavor to elucidate sex-dependent effects and optimize treatment parameters such as dosages, timing of initiation, and treatment duration. This holistic approach aims to strike a delicate balance between favorable therapeutic outcomes and potential adverse effects, thereby ushering in an era of personalized and precision medicine in HF management. 

In conclusion, continued research into inflammasome biology and its implications in HF may lead to “groundbreaking” advancements in diagnostic tools and therapeutic interventions. By continuing to unravel the “complex” mechanisms underlying inflammasome activation and its impact on HF pathogenesis, we pave the way for the development of innovative tools and effective treatments to combat this devasting disease. 

## Figures and Tables

**Table 1:** Preclinical and clinical studies on inflammasome modulation in heart failure.

Animal Studies				
Drug Name	Target	Animal Model	Main Findings	References
Colchicine	Interference with microtubule polymerization, subsequently disrupting cellular processes involved in the assembly and activation of the NLRP3 inflammasome	Spontaneously hypertensive rats	No effect on active tension or passive stiffness of the left ventricular papillary muscles.	[61]
		ATII-infused Wistar rats and cardiomyocytes	Improvement in apoptosis markers.	[62]
		I/R injury mouse model	Reduced myocardial infarct size, fibrosis, and inflammatory biomarkers. Improvement in cardiac output. No change in LVEF or diastolic function.	[63]
		AMI mouse model	Improved LVEDD, LVEF. Lower natriuretic peptides and mortality. Reduced tissue expression of inflammasome components.	[64]
		Doxorubicin-induced cardiomyopathy mouse model	Improved LVEDD, LVESD, LVEF. Decreased natriuretic peptides, fibrosis area, and expression of NLRP3 inflammasome components.	[65]
		Hypertension-induced HFpEF rat model	Ameliorated functional capacity and survival. Improved LVEDP, LV mass, BNP. Reduced myocardial inflammation and fibrosis.	[66]
MCC950	Selective NLRP3 inflammasome inhibitor through the prevention of the oligomerization process of NLRP3	Postmenopausal heart disease mouse model	Attenuated hypertrophy and improvements in myocardial relaxation, fractional shortening, and natriuretic peptides.	[67]
		Transient aortic constriction-induced pressure overload mouse model	Improved LVEF and cardiac dimensions. Diminished hypertrophy, oxidative stress, inflammation, and fibrosis.	[68]
		Isoproterenol-induced cardiomyopathy	Improvements in cardiac function, inflammation, oxidative stress, and fibrosis.	[69]
		HFpEF model	Improved pulmonary artery pressure and remodeling.	[70]
		Transient aortic constriction-induced HFpEF mouse model	Lower QTc duration, action potential duration 90, threshold for APD alternans. Decreased rate of ventricular arrhythmia induction.	[71]
CY-09	Prevention of the interaction between NLRP3 and the adapter protein ASC, which is crucial for the assembly and activation of the inflammasome	Ponatinib-induced cardiotoxicity in mice after transient aortic constriction	Improved LVEF and fractional shortening. Diminished myocardial inflammation.	[72]
Dapansutrile	Binding to the NLRP3 protein, preventing its oligomerization, which is necessary for the formation of the inflammasome complex	Non-reperfused ischemic cardiomyopathy mouse model	Improved myocardial contractile reserve, ameliorated diastolic function.	[73]
Colchicine	Interference with microtubule polymerization, subsequently disrupting cellular processes involved in the assembly and activation of the NLRP3 inflammasome	RCT of patients with stable congestive HFrEF	No change in functional capacity, hospitalization rates, or mortality. Improved LV dimensions and inflammatory markers.	[74]
Dapansutrile	Binding to the NLRP3 protein, preventing its oligomerization, which is necessary for the formation of the inflammasome complex.	Phase 1 RCT of patients with HFrEF and impaired functional capacity	Improved LVEF and exercise capacity.	[75]

ATII: angiotensin II; I/R: ischemia–reperfusion; LVEF: left ventricular ejection fraction; AMI: acute myocardial infarction; LVEDD: left ventricular end-diastolic diameter; LVESD: left ventricular end-systolic diameter; HFpEF: heart failure with preserved ejection fraction; BNP: brain natriuretic peptide; RT: randomized controlled trial; HFrEF: heart failure with reduced ejection fraction.

## Data Availability

Not applicable.
